# The DEAD-box RNA helicase RhlB is required for efficient RNA processing at low temperature in *Caulobacter*

**DOI:** 10.1128/spectrum.01934-23

**Published:** 2023-10-18

**Authors:** Hugo L. de Araújo, Beatriz A. Picinato, Alan P. R. Lorenzetti, Nisansala S. Muthunayake, I. W. Rathnayaka-Mudiyanselage, Naara M. dos Santos, Jared Schrader, Tie Koide, Marilis V. Marques

**Affiliations:** 1 Departamento de Microbiologia, Instituto de Ciências Biomédicas, Universidade de São Paulo, São Paulo, Brazil; 2 Departamento de Bioquímica e Imunologia, Faculdade de Medicina de Ribeirão Preto, Universidade de São Paulo, Ribeirão Preto, Brazil; 3 Department of Biological Sciences, Wayne State University, Detroit, Michigan, USA; Forschungszentrum Jülich GmbH, Juelich, Germany

**Keywords:** *Caulobacter crescentus*, DEAD-box RNA helicase, RNA degradosome, gene regulation

## Abstract

The RNA helicases from the DEAD-box family participate in several biochemical processes, with special relevance in the assembly of ribosome ribonucleoparticles and in RNA turnover as part of the multi-enzymatic RNA degradosome complex. *Caulobacter crescentus* has three DEAD-box RNA helicases, including RhlB, the main RNA helicase associated with the RNA degradosome. In this work, we evaluated the impact of RhlB on *C. crescentus* gene expression both at normal and low temperatures and its role in global RNA decay. The *rhlB* null mutant showed a freezing-sensitive phenotype after pre-incubation at low temperature, indicating a failure in assembling a proper cryotolerance response. A global transcriptomic profile of the wild-type and *rhlB* mutants was carried out at 30°C and 10°C and showed 73 and 477 differentially expressed genes, with the most affected gene categories being translation and posttranslational modifications. The *rhlB* mutant is more sensitive to streptonigrin, suggesting that the downregulation of iron-regulated genes may be due to a higher intracellular iron concentration. A high throughput screening for RhlB-binding RNAs identified 220 transcripts at 30°C, and global RNA decay analyses identified several mRNAs and sRNAs that displayed altered profiles of RNA decay rates. Analyses of the transcripts’ 5′-ends from the *C. crescentus rhlB* mutant and from *Pseudomonas aeruginosa* PAO1 *rhlE1* and *rhlE2* mutants confirm that in the absence of the DEAD-box RNA helicase associated with RNase E, there is an accumulation of RNA degradation intermediates.

**IMPORTANCE:**

One of the most important control points in gene regulation is RNA stability, which determines the half-life of a transcript from its transcription until its degradation. Bacteria have evolved a sophisticated multi-enzymatic complex, the RNA degradosome, which is dedicated mostly to RNA turnover. The combined activity of RNase E and the other RNA degradosome enzymes provides an efficient pipeline for the complete degradation of RNAs. The DEAD-box RNA helicases are very often found in RNA degradosomes from phylogenetically distant bacteria, confirming their importance in unwinding structured RNA for subsequent degradation. This work showed that the absence of the RNA helicase RhlB in the free-living Alphaproteobacterium *Caulobacter crescentus* causes important changes in gene expression and cell physiology. These are probably due, at least in part, to inefficient RNA processing by the RNA degradosome, particularly at low-temperature conditions.

## INTRODUCTION

RNA helicases are enzymes that act solely on RNAs, whether single-stranded, double-stranded and structured, or associated with other proteins, remodeling these molecules via an ATPase activity that is exclusively dependent on RNA (1, 2). They do not translocate on their substrates but instead unwind or remodel secondary structures locally on the RNA molecule (3). Because of these features, DEAD-box proteins participate in numerous biochemical and physiological processes, such as ribosome assembly, transcription, translation, and RNA turnover [reviewed in reference (4)].

RNA helicases are categorized into six Superfamilies according to their conserved sequences and structural elements. The DEAD-box family is the largest family within Superfamily 2, being ubiquitously distributed in all kingdoms of life, and its name is given due to the amino acid residues present in the conserved motif Asp-Glu-Ala-Asp (D-E-A-D) (5). Bacteria have variable numbers of genes encoding DEAD-box RNA helicases. In *Escherichia coli*, five of them (DeaD/CsdA, DbpA, SrmB, RhlE, and RhlB) have been described (4), of which CsdA and SrmB were originally isolated as suppressor agents of cold-sensitive mutations in genes encoding ribosomal proteins L24 and S2, respectively (6, 7). Later, they were described as important factors for the assembly of the 50S ribosomal subunit, mostly at low temperatures (8, 9). Along with SrmB and CsdA, RhlE has also been suggested to play an important role in the ribosome assembly process, by mediating the interconversion of RNA-folding intermediates to be further processed by SrmB and CsdA (10).

DEAD-box RNA helicases are often found in association with other proteins and/or with a multi-enzymatic complex called RNA degradosome. The RNA degradosome is associated with the cytoplasmic membrane in *E. coli* and *Bacillus subtilis*, acting as an RNA processing machinery whose composition varies among species (11). The *E. coli* degradosome is composed of an endoribonuclease (RNase E) associated with a phosphorolytic exoribonuclease (PNPase), a glycolytic enzyme (enolase), and a DEAD-box RNA helicase (RhlB) (12). The interactions between RNA helicases and other components of the degradosome have been reported as an important aspect of RNA processing efficiency (13, 14).

The N-terminal region of RNase E encompasses the catalytic core of the enzyme, while the C-terminal portion serves as the anchoring point for the other components of the degradosome [reviewed in reference (15)]. Although the carboxy terminus is not essential for cell viability, it has been shown that its absence affects the overall condition of the organism, changing mRNA profiles and increasing the half-life of these molecules (16, 17). RNase E has a fundamental role in several stages of RNA processing, and the degradosome’s synchronous activity of endonuclease, exonuclease, and unfolding is responsible for the efficiency of mRNA turnover (18). PNPase and RhlB can also interact between themselves even without the RNase E scaffold, affecting specific metabolic pathways such as cysteine biosynthesis (19 – 21). These interactions are an indication of the complex and dynamic regulatory network of RNA processing and degradation.

*Caulobacter* is a free-living oligotrophic bacterium, spread across humid soils and aquatic environments, associated with the plant’s rhizosphere, and well-adapted to low-temperature conditions (22, 23). Its complex cell cycle yields two dissimilar daughter cells, a sessile (stalked) cell and a natant (swarmer) cell, which require very fine tuning of regulatory mechanisms [recently reviewed in reference (24)]. Because of the rapidly changing environments in which they live, their growth and metabolism must quickly adapt. Therefore, making use of post-transcriptional regulation of gene expression allows for a rapid alteration in transcript’s stability and translation speeding up the response (25).

The *C. crescentus* NA1000 genome encodes three DEAD-box RNA helicases: RhlE, DbpA, and RhlB (26). The RNA degradosome in *C. crescentus* is associated with the cell nucleoid, and it is composed of the DEAD-box RNA helicase, RhlB, (PNPase, aconitase, and RNase D (27 – 29). All components are coupled to the C-terminal extension of the RNase E, except for RhlB, which interacts with the S1 domain of the catalytic region via its carboxy terminus portion, suggesting an evolutive adaptation to approximate the RNase E to its substrate modified by the helicase (29). As an alternative way of increasing the density of these complexes in the cell, *C. crescentus* RNase E assembles phase-separated structures (bacterial ribonucleoprotein bodies, BR-bodies) of RNA processing, providing localized sites of RNA degradation in a membrane-less structure (30). The composition of these BR-bodies may change in response to stressful conditions, according to the cell’s need for RNA processing and decay, and are enriched in mRNAs, sRNAs, and antisense RNAs, excluding highly structured RNAs such as tRNAs and rRNAs (30, 31).

The conserved association between RNase E and DEAD-box RNA helicases in bacterial RNA degradosomes indicates that the processing of RNA secondary structures by these enzymes is relevant for RNA turnover. This is even more important at low temperatures, where RNA secondary structures are stabilized, altering their half-lives and functionality. At low temperatures, the genes for the three *C. crescentus* RNA helicases are upregulated (32), and the DEAD-box helicases RhlE and DbpA, the transcription termination factor Rho, and other components are recruited to the degradosome (33). This suggests that the composition of the RNA degradosome is changed to increase the activity of unfolding RNA secondary structures at low temperatures.

This work has investigated the impact of the DEAD-box RNA helicase RhlB in *C. crescentus* gene expression profile both at 30°C and at 10°C, using global transcriptomics analyses of a *rhlB* null mutant compared to the wildtype (wt). The interaction of RhlB with specific RNAs was also evaluated by pulldown of a tagged RhlB followed by high throughput RNA sequencing, and the RNA decay rate between the two strains was determined. We have shown that RhlB has an impact on gene expression, mainly at 10°C, and is necessary for efficient and complete RNA decay by the RNA degradosome.

## RESULTS

### Role of RhlB on gene expression

Each member of the degradosome plays a distinct role in the processing and turnover of RNAs. RhlB is the main DEAD-box RNA helicase associated with this complex in *C. crescentus* (28) and may be responsible for the unwinding of transcripts for effective action of the other components, such as RNase E and PNPase. To assess the impact of RhlB on gene expression, we conducted a transcriptional profile analysis of the *rlhB* mutant strain compared to the wt strain at optimal growth temperature (30°C).

**Fig 1:**
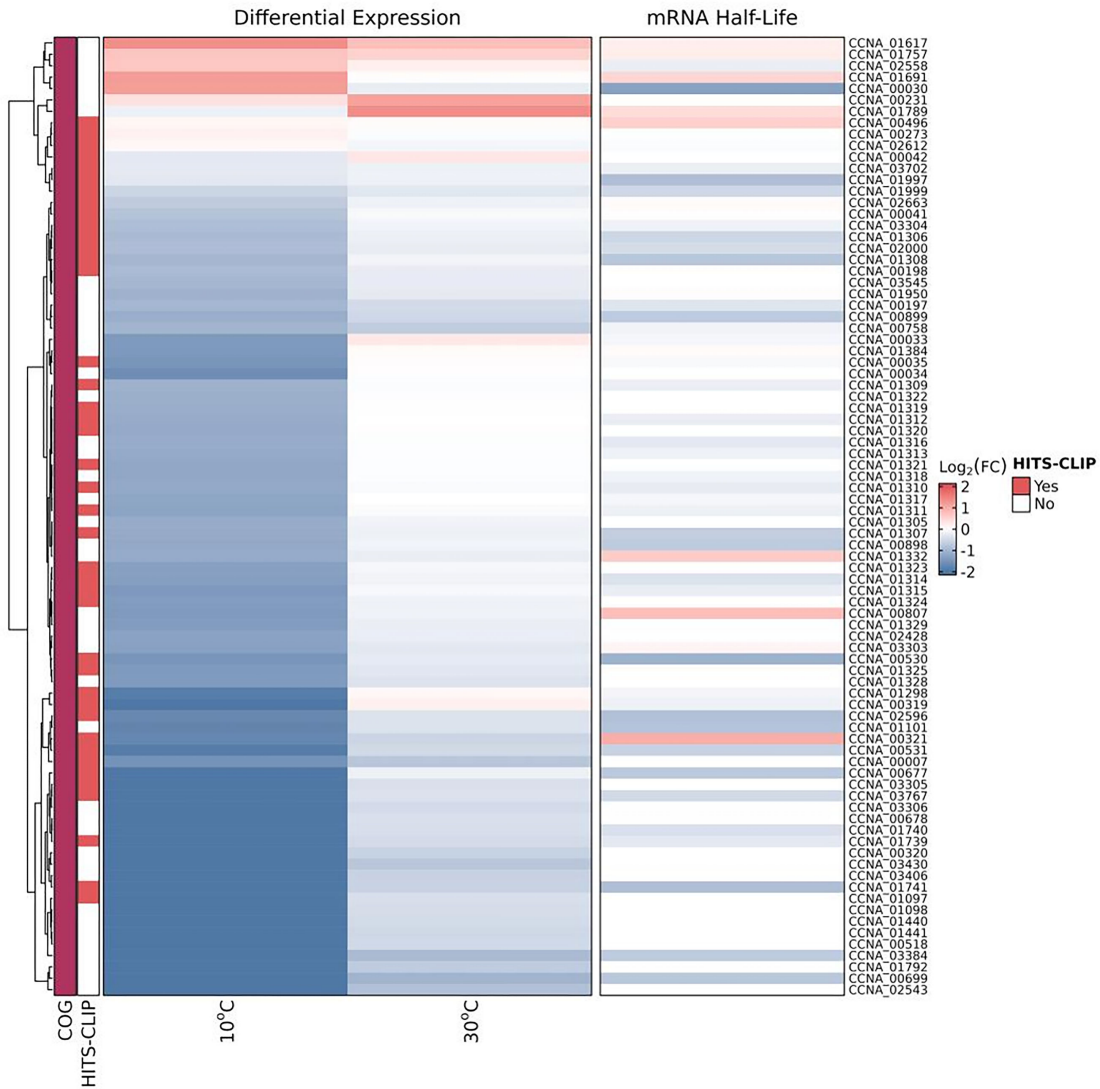
Expression, decay, and RhlB binding analyses of the DEGs belonging to the COG translation, and ribosomal structure and biogenesis. All genes that were differentially expressed in the RNA-seq or showed differential stability or whose transcript was bound by RhlB are shown. Heat map showing the DEGs determined by the RNA-seq analyses of *rhlB* x wt color-coded according to the log_2_ fold change at 30°C or at 10°C. The RNA half-life of each gene was determined after rifampicin treatment and those that showed a differential half-life are colored in the same way. RNAs that putatively bind to RhlB (enriched in the FLAG-RhlB vs RhlB strains) are indicated in red in the HITS-CLIP column. The figure was made using R package ComplexHeatmap (34).

We found that 73 (25 upregulated and 48 downregulated) genes were differentially expressed, considering a log_2_ fold change ≥1 (upregulated) or ≤1 (downregulated) (Table S3; Fig. S1). We have further analyzed the expression of selected differentially expressed genes (DEGs) by reverse transcription quantitative real-time PCR (RT-qPCR), and the results confirmed the expression pattern observed in the RNA sequencing (RNA-seq) experiments (Fig. S2), validating the RNA-seq data. A categorization of the DEGs’ functions was carried out using the Clusters of Orthologous Groups (COG) (Fig. S3). The categories overrepresented among the upregulated genes were (Fig. S3): translation, and ribosomal structure and biogenesis (Fig. 1), posttranslational modification, and energy production and conversion. Among the downregulated genes, the overrepresented categories were posttranslational modification, protein turnover, chaperones, and inorganic ion transport and metabolism.

Among the downregulated genes, the presence of several genes involved in iron metabolism is noteworthy (Table 1). The pattern of expression in the *rhlB* mutant is opposite to what was determined for these genes in response to iron availability (35, 36), i.e., genes that were upregulated in low iron were downregulated in *rhlB*, while genes that were downregulated in low iron were upregulated in *rhlB*. Interestingly, the iron-repressed genes were downregulated irrespective of their direct regulation by FUR (Table 1), suggesting that the changes in gene expression in the *rhlB* mutant were a result of changes in the intracellular iron pools.

**TABLE 1:** Genes involved in iron metabolism that were differentially expressed in the *rhlB* mutant at 30°C

Gene	log_2_ fold change (*rhlB* × wt)	padj	Product
Genes upregulated in low iron repressed by FUR^*a*^			
CCNA_00027	1.227	1.789E-11	2OG-Fe(II) oxygenase
CCNA_00028	1.615	8.283E-39	TonB-dependent receptor
CCNA_02273	1.323	7.061E-13	Glutathione peroxidase
CCNA_02274	2.159	0.006792	EF-hand domain protein
CCNA_02275	2.05	1.454E-07	ABC transporter, periplasmic component
CCNA_02277	1.884	0.002643	TonB-dependent outer membrane channel
CCNA_02452	1.584	9.112E-06	Hypothetical protein
CCNA_03155	1.241	4.164E-15	PepSY-associated transmembrane protein
CCNA_03156	1.697	5.444E-15	Putative periplasmic protein
CCNA_03157	1.719	4.305E-26	Hypothetical protein
CCNA_03371	1.089	2.692E-06	Bacterioferritin
CCNA_03807	1.237	6.772E-13	Organic solvent resistance transport system Ttg2D protein
CCNA_03808	1.215	1.738E-07	Organic solvent resistance transport system Ttg2C protein
Genes upregulated in low iron independent of FUR^*a*^			
CCNA_00059	1.427	1.124E-18	Oxygen-insensitive NADH nitroreductase
CCNA_00060	1.028	1.517E-12	Mitochondrial-type Fe-S cluster assembly protein NFU
CCNA_00722	1.096	9.184E-24	Chaperonin GroES
CCNA_01376	1.04	3.043E-08	Glutathione S-transferase
CCNA_02213	1.154	1.008E-10	Old yellow enzyme-family NADH:flavin oxidoreductase
CCNA_02341	1.324	1.596E-06	Small heat shock protein
CCNA_03097	1.594	2.167E-17	Aldo/keto reductase family protein
CCNA_03195	1.163	9.012E-13	RNA polymerase sigma factor RpoH
CCNA_03299	1.077	1.686E-10	Outer membrane protein oprM
CCNA_03806	1.094	5.532E-08	Outer membrane lipoprotein
Genes downregulated in low iron^*a*^			
CCNA_01100	1.077	7.949E-14	Acylamino-acid-releasing enzyme
CCNA_01467	3.263	0.009867	Cytochrome cbb3 oxidase subunit I ccoN

^*a*^ Pattern of expression in response to iron and Fur from references (35) and (36).

^*b*^ Values of *P* adjusted for multiple hypothesis testing using the Benjamini-Hochberg method (37).

Considering the role of RhlB in the RNA degradosome, it would be expected to observe a change in general RNA stability in the *rhlB* mutant due to the lack of helicase activity for processing structured RNAs. To evaluate this on a global scale, the half-lives of the whole transcriptome were assessed at 30°C after interrupting transcription initiation with rifampicin. Total RNA from 0, 1, 3, and 9 min after rifampicin treatment was sequenced to measure the half-lives of mRNAs in the wt vs *rhlB* mutant. It is important to note that with these time points, we were not able to determine the half-lives for mRNAs that are very unstable (half-lives < ~1 min are not calculable) or also very stable (half-lives > ~15 min are not calculable). In addition, as the RNA-seq libraries were made by shearing the RNA to 50 nt fragments by base hydrolysis, these samples contain a mixture of fragments originating from full-length mRNAs as well as from partially degraded mRNAs. We could not determine a measurable difference between the two strains in bulk half-life, because the replicates of the RhlB mutant showed significant variation. The results showed that 24 transcripts were more stable in the mutant, while 110 mRNAs were less stable, as well as 25 sRNAs whose half-life was significantly altered between the strains (Table S4). However, these results did not show a correlation with the differentially expressed genes identified in the RNA-seq analyses, potentially due to the lower number of mRNAs measured in the half-life experiment.

As a strategy to identify the transcripts interacting directly with RhlB, we performed co-immunoprecipitation assays using a FLAG-tagged RhlB, followed by high throughput sequencing of the cross-linked RNAs (HITS-CLIP). A transcript was considered RhlB-bound if it was enriched in the FLAG-RhlB library compared to the untagged RhlB pulldowns by peak-calling analysis. However, it must be noticed that as RhlB binds RNase E in the RNA degradosome, there is a possibility that RNA pulled down in the HITS-CLIP experiment is bound by the RhlB-RNase E complex. A total of 220 RNA targets were identified (Table S5), five of which were also downregulated in the *rhlB* strain. In summary, the absence of RhlB caused a mild effect on global gene expression at 30°C, and there was not a large overlap between DEGs and RhlB-bound transcripts, suggesting most of this effect could be indirect. Moreover, RhlB may bind different sets of RNA when in complex with RNase E or free in the cell, which could account for the variation seen in the data sets.

### The *rhlB* mutant is impaired for cryotolerance

**Fig 2:**
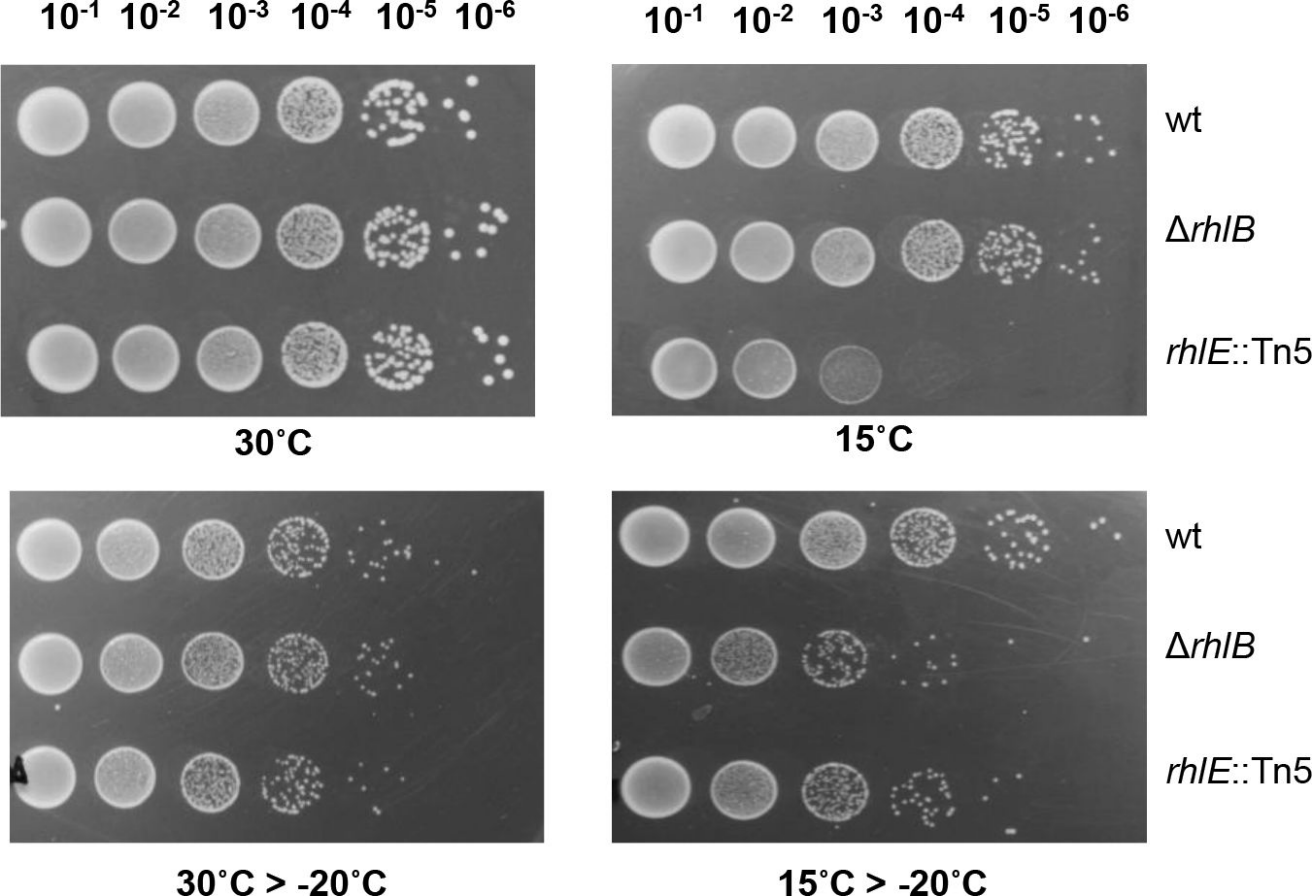
Viability tests of the NA1000, ∆*rhlB,* and *rhlE*::Tn5 strains at low temperature and freezing. The wt (NA1000), ∆*rhlB,* and *rhlE*::Tn5 strains were grown in PYE medium until mid-log phase at optimal temperature condition (30°C), after a 2 h cold shock (15°C), after 24 h freezing at −20°C with no pre-incubation (30°C > −20°C), and after 24 h freezing at −20°C with a 2 h pre-incubation at 15°C (15°C > −20°C). The cultures were subject to serial dilutions and plated in PYE. The figure is representative of two independent biological experiments with duplicates.

The Δ*rhlB* mutant strain shows a mild growth defect at 15°C, and this may be compensated by overexpressing the *rhlE* gene, suggesting that the phenotype is due to a lack of sufficient helicase activity (33). To test whether the lack of *rhlB* could cause a more pronounced phenotype in more severe conditions, we tested the strains for resistance to freezing. The NA1000 (wt), *rhlB*, and *rhlE* strains were grown either at 30°C or after 2 h at 15°C and were further transferred to a −20°C freezer and incubated for 24 h prior to plating for CFU counting. As shown in Fig. 2, all strains have similar growth at 30°C, but the *rhlE* mutant viability is severely affected after 2 h at 15°C. The *rhlB* mutant shows slightly smaller colonies at 15°C, but no loss of viability. After 24 h freezing, no difference in viability was observed among the cultures previously grown at 30°C, all showing about a 10-fold reduction in viability. However, the *rhlB* and *rhlE* mutants showed a significant reduction in viable cell counts than the wt in cultures pre-incubated at 15°C for 2 h before freezing (Fig. 2). These mutants did not show any growth phenotype when exposed to high osmolarity at 30°C (85 mM NaCl or 150 mM sucrose) or oxidative stress (3% H_2_O_2_) (Fig. S4). This indicates that the freezing protection generated by cold adaptation is inefficient in the absence of these RNA helicases and that the role of these enzymes is particularly important in response to this stress.

### RhlB has a large impact on gene expression at low temperature

The *rhlB* gene is induced at low temperature (32) and the growth phenotype of the *rhlB* mutant in response to freezing prompted us to evaluate whether the lack of RhlB would have an impact on gene expression that could explain the impaired cryotolerance. Therefore, a global transcriptome profile of *rhlB* mutant × wt was obtained from cells incubated for 2 h at 10°C, which is a condition that elicits the cold stress response (32). A larger number of DEGs was observed (161 upregulated and 317 downregulated) when compared to 30°C (Table S3). Fourteen DEGs showed similar profiles both at 30°C and 10°C, with four being upregulated (CCNA_02781, 02791, 03709, and 03973), and ten downregulated (*groES*, CCNA_00699, 01585, 01770, 02273, 02341, 03371, 03587, 03666, and 03769) in the *rhlB* mutant. Six genes showed opposite expression profiles between temperatures (CCNA_00882, 02452, 03155, R0001, R0037, and R0058).

It is noteworthy that after the 2 h cold treatment, the number of DEGs in the *rhlB* mutant increased about sixfold, belonging mainly to the COGs, the inorganic ion transport and metabolism; transcription; amino acid transport and metabolism; translation, ribosomal structure and biogenesis; and posttranslational modification, protein turnover, chaperones (Table S3). Interestingly, the products of several genes related to translation, ribosomal structure and biogenesis were also identified as RhlB-bound (Table S5) and were also downregulated at 10°C, indicating a prominent role of RhlB in this category, mainly at low temperature (Fig. 1). Moreover, the range of differential expression values was also higher at 10°C, of approximately 9- to 18-fold for the most differentially expressed genes. These results indicate that the absence of RhlB impacts gene expression more intensely at low temperature, probably due to changes in the transcripts’ turnover rates. In fact, 32 transcripts that showed differential stability in the half-life experiment were differentially expressed at 10°C, with a correspondence between the lower expression and the shorter half-lives (Tables S3 and S4). The increase in the number of DEGs at 10° including the lower CspA (4-fold) and CspB (2.2-fold) expression (Table S3) may help explain the growth defect and lack of cryotolerance observed for the *rhlB* mutant.

**Fig 3:**
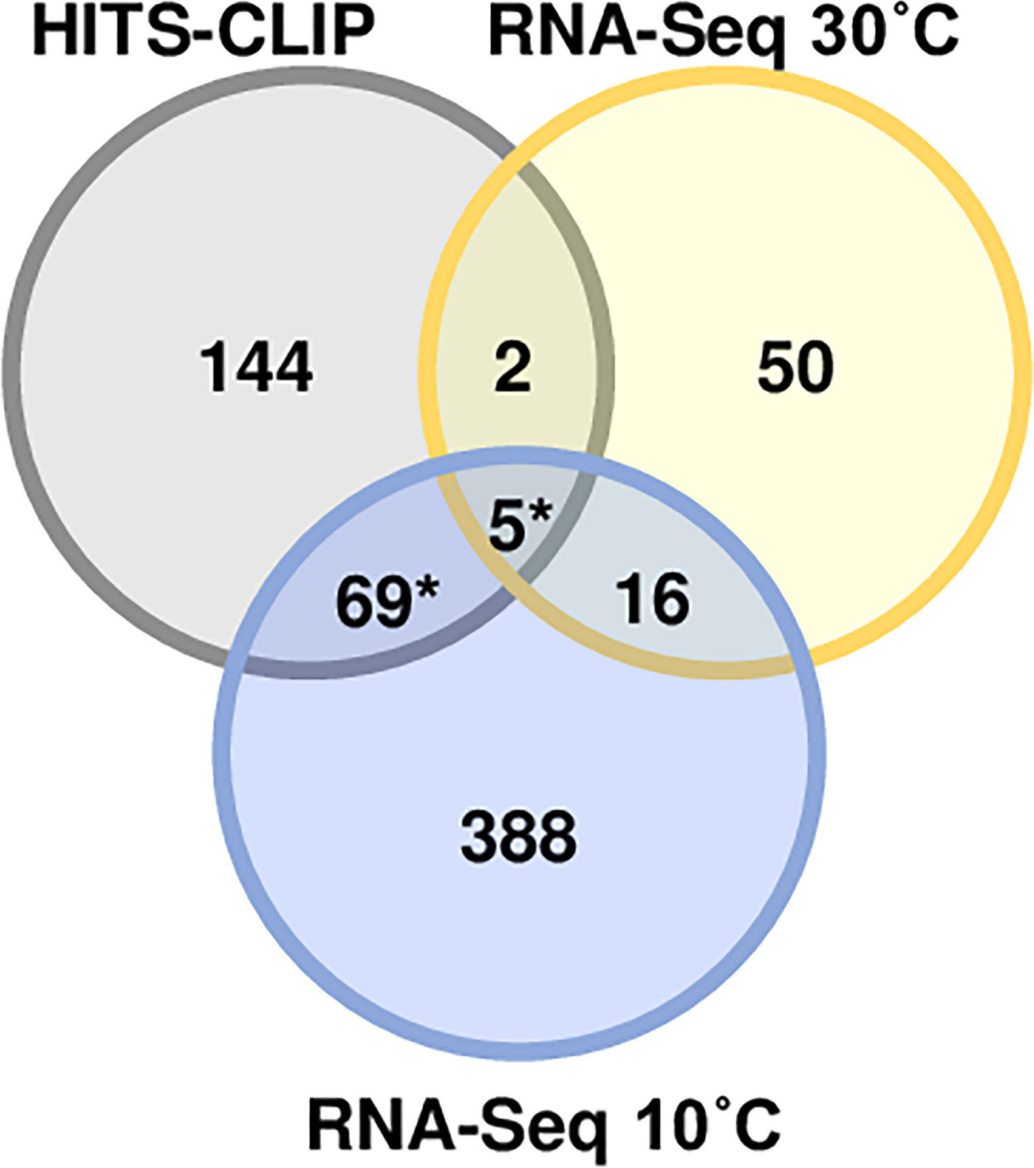
Comparison of the RNA-seq and the HITS-CLIP experiments. Venn diagram representing the number of differentially expressed genes in both RNA-seq analyses (at 30°C and 10°C) and identified as binding to RhlB in the HITS-CLIP experiment. Asterisk, significant according to the Benjamini-Hochberg method (37).

**Fig 4:**
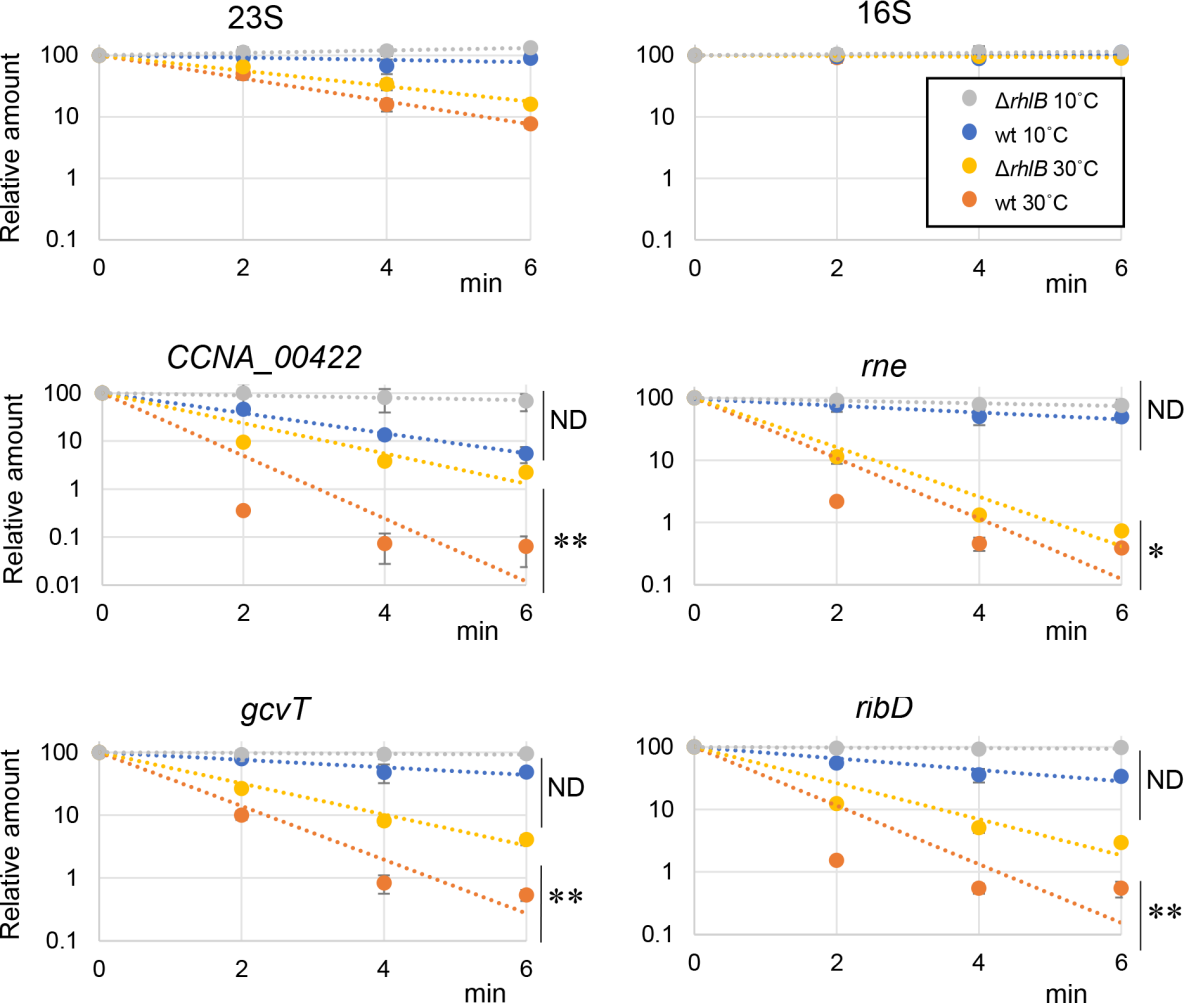
mRNA decay of selected mRNAs. The amounts of four mRNAs (*rne, gcvT, ribD,* and CCNA_00422), identified either as a DEG or as bound to RhlB in the HITS-CLIP, were measured by RT-qPCR for up to 6 min after the addition of rifampicin. The decays were determined in both the wt (NA1000) and ∆*rhlB* strains at 30°C and after 2 h at 10°C, relative to the amount at time 0, using biological triplicates. rRNAs 16S and 23S were used as controls. *t*-test: *P < 0.05, **P < 0.01; and ND, not applicable (half-life cannot be calculated as the mRNA is stable for longer than our longest time-point).

When comparing the HITS-CLIP results (set of 216 genes bound to RhlB, except for the rRNA) to the cold stress transcriptome (478 genes), we observed a significant overlap of 74 transcripts (*P* = 1.82 × 10^−19^, Fig. S5), with only two of them being upregulated in the *rhlB* strain (Fig. 3). We selected four genes (*rne*, *gcvT*, *ribD,* and *CCNA_00422*) to assess their decay rate between the wt and *rhlB* mutant strains at both temperatures (Fig. 4). All of them showed a lower decay rate at 30°C and at 10°C in the mutant when compared to the wt. However, at 10°C, we could not have a statistical validation due to the extreme stabilization of the transcripts at this temperature and the timescale of the experiment.

**Fig 5:**
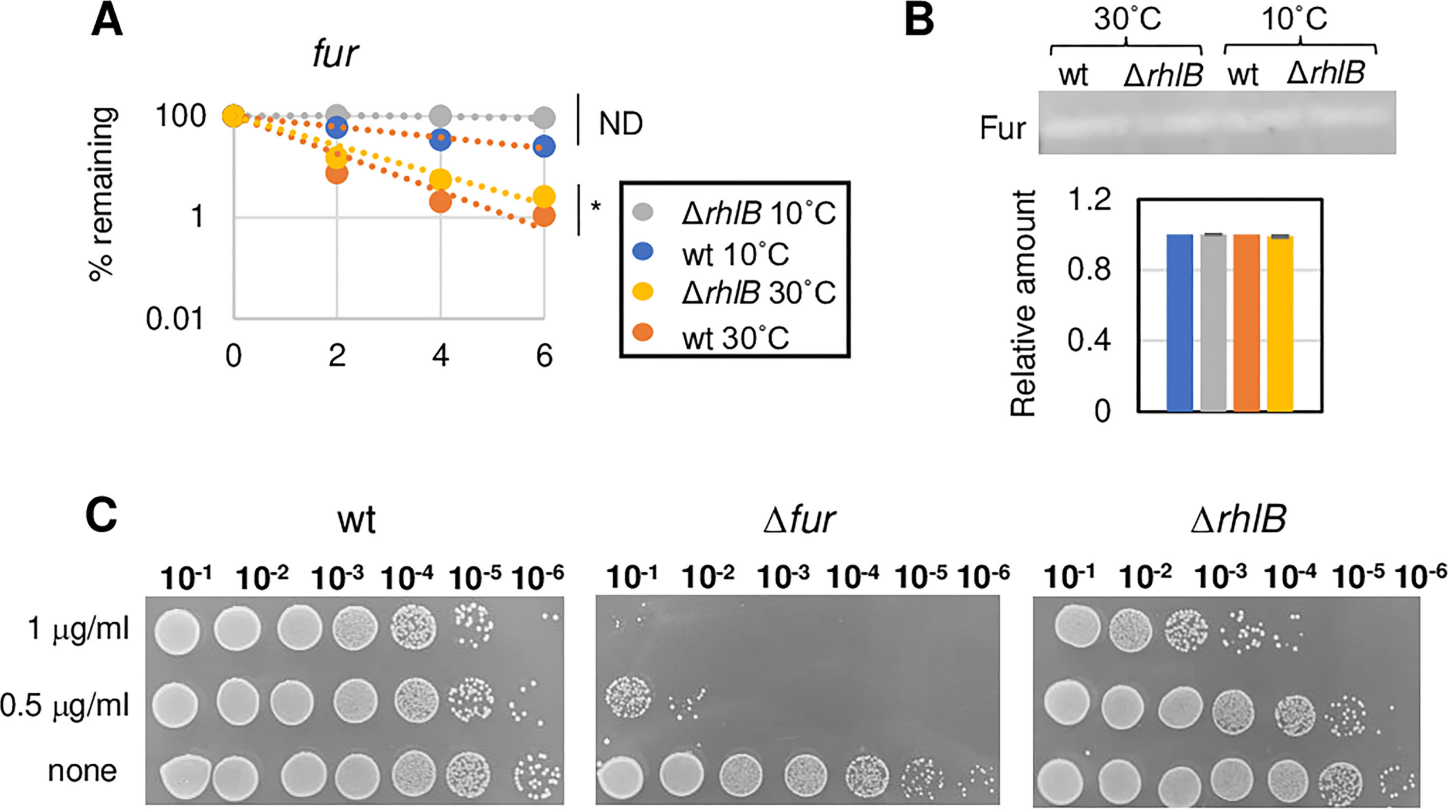
Effect of the lack of RhlB on iron homeostasis. (**A**) Quantification of Fur transcript in the ∆*rhlB* strain. The decay rate for *fur* was measured in the wt (NA1000) and in the ∆*rhlB* strains at 30°C and after 2 h at 10°C, using biological duplicates. *t*-test: *P < 0.01 and ND, not applicable. (**B**) Determination of Fur levels by Western blotting. Equal amounts of protein extracts from each strain at both temperatures were separated by SDS-PAGE, transferred to a nitrocellulose filter, and this was incubated with anti-Fur antiserum at a 1:1,000 dilution (39). The bands were developed using a secondary anti-rabbit IgG conjugated with Alexa Fluor 488. Quantification of Fur levels was obtained by pixel density using ImageJ in both strains at both temperatures previously cited. (**C**) Streptonigrin resistance of the *fur* and *rhlB* mutants. Cultures of each strain were incubated with 0 (control), 0.5, or 1 µM streptonigrin for 24 h, and serial dilutions were plated on PYE medium. The plates were incubated for 3 days at 30°C. These images are representative of six independent biological replicates.

Interestingly, several genes from the iron stimulon were downregulated at 30°C in the *rhlB* mutant (Table 1) but their transcripts did not co-precipitate with FLAG-RhlB. Since *fur* itself was not differentially expressed in our RNA-seq analysis, we tested if this effect was related to increased Fur levels. Levels of *fur* mRNA and Fur protein at both temperatures were measured in both strains, showing that although the transcript is stabilized after 2 h at 10°C, the protein levels seem unaffected either by the absence of RhlB or by cold stress (Fig. 5A and B). The downregulation of genes belonging to the Fur regulon has also been reported for the *E. coli rhlB* mutant, without alteration of *fur* transcript levels as well (16), indicating that there must be a conserved regulatory pathway. To test whether this effect on the *rhlB* mutant was due to a higher intracellular iron concentration, a streptonigrin sensitivity test was carried out, as antibiotic killing depends on the intracellular concentration of iron. As controls, we used the wt NA1000 strain and the *fur* mutant, which is highly sensitive to the antibiotic drug because of the deregulated iron homeostasis (38). The results showed (Fig. 5C) that the *rhlB* mutant is slightly more sensitive to streptonigrin than the wt, indicating that there is a higher concentration of intracellular iron that could generate the observed repression of the Fur regulon.

### RhlB is required for complete RNA processing

**Fig 6:**
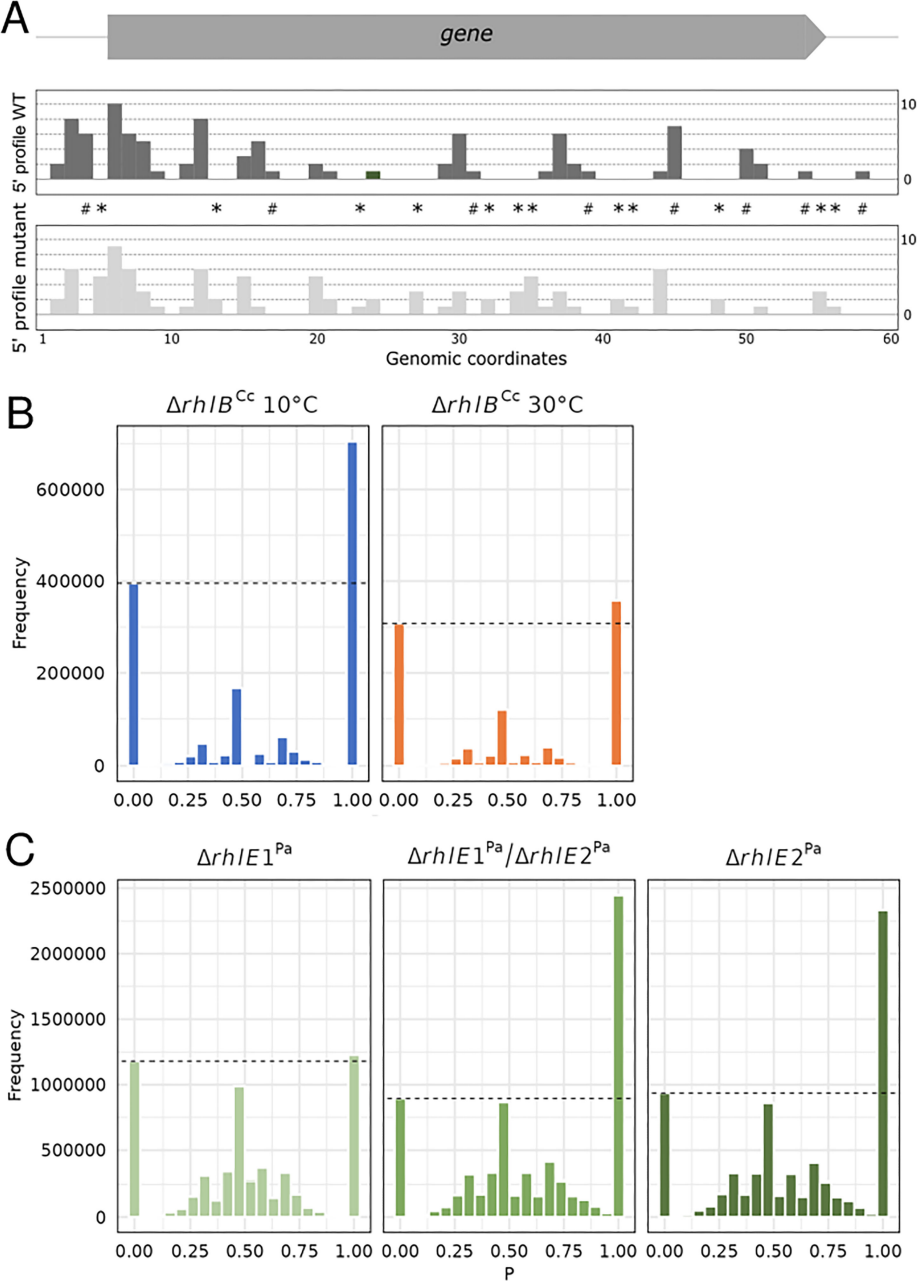
Evidence of RNA decay intermediates accumulation in the ∆*rhlB* strain at the genome level. (A) Scheme of 5′ profiles for a hypothetical gene (gray arrow, top) for both the wild-type (dark gray) and mutant strains (light gray). Colored bars in the 5′ profiles represent how many sequenced fragments (*y*-axis) started at that genomic position (*x*-axis). The proportion *P* of 5′ end counts of sequenced fragments in the mutant strain relative to the total of 5′ end counts of both mutant and wild-type strains was calculated for every genomic position. Genomic positions, where *P* = 0, i.e., no reads starting at that position in the mutant, and where *P* = 1, i.e., no reads starting at that position in the wild-type strain, are shown by # and *, respectively. (B) Histogram of values of *P* calculated for the *C. crescentus* whole genome (∆*rhlB* vs wt) at 10°C and 30°C. This analysis of fragmentation excess in RNA-seq data showed many more 5′ ends of RNA fragments sequenced exclusively in *rhlB* mutant strain (*P* = 1) relative to a background level of wild-type exclusive reads (*P* = 0) at 10°C at the genome level (BER = 7.6 × 10^−5^). At 30°C, the observed difference is not significant (BER = 0.37). (C) Histogram of values of *P* calculated for the *P. aeruginosa* PAO1 whole genome for ∆*rhlE1*, ∆*rhlE2,* and ∆*rhlE1*∆*rhlE2* mutants relative to the wild-type strain using publicly available RNA-seq data (40). This analysis showed more 5′ ends of RNA fragments sequenced in *rhlE2* and *rhlE1/rhlE2* mutant strains (*P* = 1) relative to a background level of wild-type exclusive reads (*P* = 0) at the genome level (BER equal to 3.5 × 10^−4^ and 8.1 × 10^−5^, respectively). In *rhlE1,* the observed difference is not significant (BER = 0.92).

The global transcriptome was further analyzed to verify if the *rhlB* mutant strain showed impaired processing capabilities relative to the wt strain. There is a background underlying the rate of fragmentation intrinsic to the sequencing protocol; therefore, our interest was to verify an excess of fragments aligned considering the mutant strain. An overview of such fragmentation is obtained by the histogram of the proportion of aligned 5′ read ends of sequenced fragments of the mutant relative to the wt strain. Of special interest are the read ends that are seen in one strain but not seen in the other. The fraction *P* of fragment starts seen in the mutant divided by the sum of fragment starts seen in both strains for all genomic positions was calculated and its histogram is shown in Fig. 6. At 10°C, the frequency of read start positions that exist only on the *rhlB* mutant (*P* = 1) is clearly larger than the opposite (*P* = 0, reads only in wild-type strain): there are approximately 78% more genomic positions that have *P* = 1 relative to *P* = 0 (Bayesian error rate, BER = 7.6 × 10^−5^, Fig. 6B, left panel). The amount of reads at *P* = 0 is probably due to random fragmentation and the difference in frequency at *P* = 1 is a fragmentation excess associated with the RhlB absence.

Results from 10°C and 30°C vary markedly. The mutant strain clearly cannot eliminate cleaved RNAs as efficiently as the wt strain at low temperature. The effect practically disappears at 30°C since the excess of mutant-exclusive fragment signals is minimal: there is only a 16% increase of genomic positions that have *P* = 1 relative to *P* = 0 (BER = 0.37, Fig. 6B, right panel). We could not see the same excess effect comparing RNA-seq libraries from another *C. crescentus* mutant to the wt strain, and different samples from the undersampling approach (see Materials and Methods) generated almost identical results (Fig. S6).

To evaluate if this increase of 5′-ends is related to the absence of a DEAD-box RNA helicase in the RNA degradosome complex, we used the same method to analyze previously published RNA-seq data from *P. aeruginosa* RhlE mutants (*rhlE1*, *rhlE2,* and double *rhlE1*/*rhlE2*) (40). RhlE2 is a DEAD-box RNA helicase that binds to RNase E via its C-terminal region and is associated with *P. aeruginosa* RNA degradosome, while RhlE1 does not. While both *rhlE1* and *rhlE2* mutants are defective in cold adaptation, the lack of *rhlE2* presents additional phenotypes regarding motility, biofilm formation, and virulence. We have analyzed the data of the three *P. aeruginosa* RhlE deletion mutants and it can be seen in Fig. 6C that the *rhlE2* mutation (single or double) causes an accumulation of 5′-ends when compared to the wt strain PAO1 (BER equal to 3.5 × 10^−4^ and 8.1 × 10^−5^, respectively), while the absence of *rhlE1* does not (BER = 0.92). These results validate our analysis of the *C. crescentus rhlB* mutant and confirm that in the absence of the DEAD-box RNA helicase associated with RNase E, there is an accumulation of RNA degradation intermediates and that RNA processing is severely affected likely due to inefficient degradation of structured molecules either by RNAse E and/or PNPase.

## DISCUSSION

The maintenance of cell homeostasis and stress responses are controlled by a complex and multilayered regulatory network of gene expression, where RNA processing and turnover play an important role. The modulation of RNA secondary structure by DEAD-box RNA helicases and their ubiquitous presence in the RNA degradosomes from several bacteria indicate their important cellular role. The RNA degradosome composition varies among bacteria, and the role of each component may differ according to the combination of enzymes and the bacterium’s lifestyle (11). In this study, we attempted to elucidate the importance of the RNA helicase RhlB in the free-living bacterium *C. crescentus*, by comparing a null *rhlB* mutant strain to its isogenic wildtype. The absence of RhlB was previously shown not to affect the binding of the other RNA degradosome components, so the observed effect would be only due to the lack of RhlB activity (33).

While RhlB is the main DEAD-box helicase associated with the degradosome at 30°C (28), other RNA helicases such as RhlE and DbpA probably have a prominent role at low temperature, since they are highly induced in this condition and were shown to associate with the RNA degradosome (22, 32, 33). This induction of RNA helicase expression in low temperature has also been reported for other bacterial species (41, 42), confirming the necessary increase in RNA unwinding activity for cold adaptation. The cold phenotype of the *rhlB* mutant is less severe than that of *rhlE* knockout (33), which is also observed in *Yersinia pseudotuberculosis,* where the *rhlE* mutant shows a worse growth defect when compared to the *rhlB* mutant at low temperature (43). Although the phenotype of the *rhlB* mutant is not as severe at low temperature, the *rhlB* gene is also upregulated in response to cold stress (32), suggesting that it may have a role in adaptation in this condition. We showed that the lack of RhlB impairs the mounting of an efficient cryotolerance response that protects against the effects of freezing (Fig. 2), suggesting that gene expression of protective factors is compromised. In fact, two of the cold shock protein genes, *cspA* and *cspB*, were downregulated in the *rhlB* mutant at 10°C. These genes were shown to be required for growth at low temperature, and the double *cspA/cspB* mutant presented a severe cold-sensitive phenotype, showing no growth after 24 h at 10°C (44). The expression of other proteins important for cold adaptation could also be affected, leading to the observed freezing-sensitive phenotype.

While in *E. coli,* the RNA degradosome is associated with the cytoplasmic membrane, and that interaction seems to be mediated by RhlB to some extent (45 – 47), in *C. crescentus*, RNase E does not have a membrane-targeting sequence. Instead, the RNA degradosome forms ribonucleoprotein bodies in the nucleoid region (27, 30, 31). The link between the degradosome and the ribosome assembly seems to be conserved since the 23S, 16S, and 5S rRNAs, transcripts encoding ribosomal proteins, and other components of the translation apparatus were found to be bound to RhlB and/or differentially expressed in the *rhlB* strain, especially at 10°C (Fig. 1; Tables S3 and S5). We observed an association between the RNAs directly bound by RhlB and their downregulation, indicating a causal effect, although we cannot rule out an indirect effect. DEAD-box RNA helicases are important for the assembly of the ribonucleoprotein complexes to generate the mature ribosome (10, 48, 49). If RhlB is associated with the assembly of the translation apparatus and/or unwinding mRNAs, the effect on the transcripts could be due to these transcripts not being efficiently translated.

A very intriguing result was the downregulation of genes related to iron metabolism in the *rhlB* mutant at 30°C (Table 1). These genes were previously identified as induced by iron depletion, some, but not all, in a FUR-dependent manner (35, 36, 39). The same phenomenon was observed for the *E. coli rhlB* mutant, but not for a strain carrying a truncated version of RNase E, suggesting this is mediated by the complete RNA degradosome (16). This phenotype could result from the failure to sense iron levels accurately, leading to increased intracellular iron levels, which was confirmed in *C. crescentus* by the sensitivity of the *rhlB* strain to streptonigrin (Fig. 5C). In *E. coli*, the downregulated genes were regulated by FUR either directly or indirectly via the small regulatory RNA RyhB, but to date, no sRNA functionally equivalent to *ryhB* has been described in *Caulobacter*. Notably, the iron-regulated genes are also severely downregulated when *C. crescentus* is at low temperature (32), and we cannot exclude the possibility of posttranscriptional mechanisms of regulation. A very interesting aspect is that aconitase is part of the *C. crescentus* RNA degradosome, and it has been reported in other organisms to have a regulatory role. When levels of iron are low and aconitase is in the apo form (without the Fe-S group), it binds to RNA and may affect its decay rates (50). An intriguing possibility is that inefficient RNA degradation in the *rhlB* mutant could account for the lower activity of aconitase-mediated RNA decay, resulting in an imbalance in iron homeostasis. We are currently investigating the role of aconitase in regulating iron homeostasis and its relationship with the RNA degradosome.

Interestingly, the global analysis of mRNA half-lives in the *rhlB* mutant did not show a large stabilization effect on transcripts (Table S4). The same was described for *E. coli*, where the *rhlB* mutant showed just a mild increase in the median half-life (16). It was proposed that RhlB is only necessary for RNase E and PNPase to function properly when they encounter highly structured transcripts (14). In *C. crescentus*, most DEGs were downregulated in the *rhlB* mutant at both temperatures, suggesting that the degradosome without RhlB can still degrade most RNAs, even with the stabilization of transcripts at 10°C. However, when we observe the positions of 5′-ends from sequenced fragments of both strains, we see a large increase in decay intermediates at 10°C (Fig. 6). Importantly, rif-seq mRNA half-lives are based upon the measure of small fragments of ~50 nt, which includes both fragments liberated from full-length mRNAs or from mRNA decay intermediates. The mRNA decay measurement follows the loss of RNA mass, which does not necessarily correlate with the functional inactivation of the full-length transcript.

Our results suggest that there may be a decoupling of initial cleavage by RNase E and subsequent exonucleolytic decay facilitated by degradosome-associated exonucleases in the *rhlB* mutant. This effect could be due to the inefficient processing of the transcripts by the PNPase and RNase D, as a similar effect was observed for a *C. crescentus* mutant where the binding sites for these enzymes were deleted from RNase E (31). This was more pronounced at 10°C, indicating that the RNA degradation is less efficient in the absence of RhlB, and this is increased when the transcripts are more structured at low temperature. In *E. coli*, mutants lacking RhlB and PNPase were shown to accumulate mRNA decay fragments that contained repeated extragenic palindrome sequences (51) and in an RhlB-activated degradation of structured RNA by PNPase (52).

In conclusion, our results indicate that RhlB plays a role in *Caulobacter* gene regulation, particularly at low temperature, likely a result of the helicase action over structured RNAs. The absence of RhlB affects the expression of genes involved in iron metabolism, translation, and cold adaptation, leading to a deregulated iron homeostasis and failure to mount an effective cryoprotective response, and it is necessary for the complete processing of decay intermediates. However, this was not likely due to a comprehensive effect on transcript stabilization, since no major changes in half-lives were observed in the *rhlB* mutant. This is a result of the helicase action over structured RNAs, but we cannot exclude the possibility of a direct RhlB-mediated increase in the efficiency of the two degradosome exoribonucleases PNPase and RNase D.

## MATERIALS AND METHODS

### Strains and growth conditions

The *C. crescentus* and *E. coli* strains used in this work are described in Table S1. *C. crescentus* NA1000 (53) was used as the wt strain to compare with the isogenic *rhlB* mutant strain in the transcriptome analysis. *C. crescentus* cultures were grown at either 30°C or 10°C with agitation in M2 minimal medium or peptone-yeast extract (PYE) complex medium (54). *E. coli* was grown in a lysogeny broth medium (Sigma-Aldrich). The strains harboring the pBV-MCS4 vector (55) were grown in media containing gentamycin: 0.5 µg/mL for *C. crescentus*; 15 µg/mL for *E. coli*. Vanillate (0.5 mM, Sigma-Aldrich) was also added to the *C. crescentus* cultures when necessary to induce the expression of the *rhlB* gene from the pBV-MCS4 vector.

### Phenotypic tests

The NA1000 and Δ*rhlB* and *rhlE*::Tn5 strains were grown in PYE at 30°C overnight, cultures were diluted for optical density at 600 nm (OD_600nm_) = 0.1 and incubated until OD_600nm_= 0.3, when small differences among the samples were adjusted to give equal numbers of cells/mL. Aliquots from each culture were: (i) plated on PYE plates and incubated at 30°C or 15°C for the colony-forming unit (CFU) counting; (ii) transferred to a −20°C freezer for 24 h and plated on PYE plates at 30°C for CFU counting; (iii) incubated at 15°C for 2 h and then transferred to a −20°C freezer for 24 hr and on PYE plates at 30°C for CFU counting.

The streptonigrin sensitivity test (56, 57) was carried out using the NA1000, Δ*rhlB,* and Δ*fur* strains. Cultures were incubated at 30°C in PYE for 16 h. The OD_600nm_ of each culture was adjusted for 0.1 and incubated with either 0, 0.5, or 1.0 mg/mL Streptonigrin (Sigma-Aldrich S1014) for 24 h at 30°C. Aliquots were then plated and incubated for 48 hr at 30°C to determine CFU.

### Total RNA extraction and sequencing

Growth at low-temperature conditions was obtained by transferring cultures (OD_600nm_ = 0.5) grown in M2 medium in 50 mL Erlenmeyer flasks at 30°C to a shaker at 10°C and further incubating them with agitation at 250 rpm for 2 h. Aliquots from three independent biological replicates grown at 30°C or 10°C in the same batch of M2 medium were collected and maintained at −80°C until RNA extraction.

Total RNA was extracted from 2 mL aliquots using the RNeasy Mini kit (Qiagen) and RNA concentration and integrity were verified with Agilent 2100 Bioanalyzer (Agilent Technologies, Waldbroon, Germany). rRNA depletion and cDNA libraries were prepared using the Illumina Stranded Total RNA Ligation with RiboZero Plus kit (Illumina), essentially as described in reference (32). The concentration and quality of libraries were analyzed using Bioanalyzer (kit DNA 1000), and sequencing was carried out using the NextSeq 500/550 Mid Output kit v2.0 (150 cycles) (Illumina) in an Illumina NextSeq 500 (Illumina) instrument.

### Reverse transcription quantitative real-time PCR

For the RT-qPCR assays, cultures were grown in M2, and total RNA was extracted from 1 mL aliquots with TRIzol RNA (Invitrogen Life Technologies), following the manufacturer’s instructions. RNA concentration after extraction was determined by OD 260/280 nm ratio and integrity was confirmed by electrophoresis.

Samples were processed as described previously (32). Briefly, RNA was treated with DNase I (Invitrogen), and DNA degradation was confirmed by PCR using primers for the *rho* gene (Table S2). Synthesis of cDNA was performed using the SuperScript III first-strand synthesis kit (Invitrogen). RT-qPCR was performed using Power SYBR Green and PCR Master Mix (Applied Biosystems), using CCNA_02070 as the internal gene for reference control. Tested RNAs were amplified using primer pairs described in Table S2, and reactions were performed in the StepOnePlus Real-Time PCR System (ThermoFischer Scientific). Relative change in the normalized expression of each gene was calculated by the 2^-ΔΔCt^ relative expression quantification method (58).

### mRNA decay

*C. crescentus* NA1000 and Δ*rhlB* were grown in 10 mL M2 medium with agitation until OD_600nm_= 0.5 at 30°C, and after a 2-h cold shock at 10°C. To determine the stability of specific mRNAs, 1 mL aliquots were taken from three independent growths in each condition. Aliquots were taken before (*t*_0_) the addition of rifampicin (200 µg/mL) and after 2 (*t*_2_), 4 (*t*_4_), and 6 (*t*_6_) min at both temperatures. Samples were centrifuged at 12,000 rpm for 1 min, and pellets were resuspended with TRIzol RNA (Invitrogen Life Technologies) and immediately frozen in dry ice with ethanol. RNA extraction and cDNA synthesis were performed as described above. RT-qPCR was performed using primer pairs to amplify each gene (Table S2). To determine the mRNA decay rate in each condition, the *C*_*T*_ of the *t*_0_ time point was used as a reference to calculate the relative mRNA levels (Δ*C*_*T*_), from which we calculated the expression ratio (2^-ΔΔCt^) for each time point.

### Construction of a strain carrying a FLAG-tagged RhlB

We used the QuikChange Lightning Site-Directed Mutagenesis kit (Agilent) to introduce the M2 (FLAG) coding sequence (primers FLAG-RhlB Fw and Rv) in a previous construction of the *rhlB* gene in the pBV-MCS4 plasmid (33). The correct introduction of the epitope was confirmed by DNA sequencing and Western blotting using a monoclonal antiFLAG M2 antibody (Sigma-Aldrich). The plasmid was used to transform *E. coli* S17-1 and subsequently was transferred to the *rhlB* mutant strain by conjugation.

### High-throughput sequencing of RNA by cross-linking immunoprecipitation (HITS-CLIP)

RhlB RNA targets were identified via co-immunoprecipitation assays using the *rhlB* mutant strain carrying the pBV FLAG-RhlB plasmid as previously described (59, 60). Briefly, *C. crescentus* strains Δ*rhlB* pBV-FLAG-RhlB and Δ*rhlB* pBV-RhlB (without the epitope) were grown in 800 mL of M2 medium with gentamicin with agitation at 30°C until OD_600nm_= 0.3. Vanillate (0.5 mM) was added, and cultures were incubated for two more hours, reaching a final OD_600nm_ of ~0.5. Formaldehyde was then added to a 0.5% concentration for crosslinking, and cultures were incubated at 30°C for 10 min. RNA extractions were performed using TRIzol RNA (Invitrogen Life Technologies), and the purified RNAs were resuspended with 16 µL diethyl pyrocarbonate (DEPC) water. Isolated RNAs were processed as reported in the RNA-seq section.

RNA samples from two independent biological replicates from the pBV-RhlB control strain and three RNA independent biological replicates from the pBV FLAG-RhlB strain were submitted to high throughput sequencing. One microgram of RNA from each sample was used to prepare the cDNA libraries using the Illumina Stranded Total RNA Ligation with RiboZero Plus kit (Illumina), skipping the ribosomal RNA depletion step.

### Analysis of mRNA decay rates by Rif-seq

*Caulobacter crescentus* NA1000 cells and delta *rhlB* cells were grown in an M2G medium overnight. The next day, after diluting the cultures, the mid-log-phase cultures (OD_600nm_ = 0.3–0.5) at 30°C were treated with 10 mg/mL rifampicin resulting in a final concentration of 200 µg/mL in the culture. Just prior to rifampicin addition, 1 mL of culture (0 min) was removed and added to 2 mL of RNAprotect Bacteria Reagent (QIAGEN), vortexed, and incubated at room temperature for 5 min. This step was repeated at 1-, 3-, and 9-min post-rifampicin addition. The cells were pelleted at 20,000 × *g* for 1 min in 1.5 mL microtubes to prepare for RNA extraction.

Pelleted cells were resuspended in 1 mL of pre-warmed (to 65˚C) TRizol Reagent (Ambion) and incubated for 10 min at 65°C. Two hundred microliter of chloroform wasadded to the samples and the tubes were gently inverted a few times. Then, the samples were incubated at room temperature for 5 min and spun at 20,000 × *g* for 10 min. The supernatant was transferred to siliconized tubes and 500 µL of chloroform was added, vortexed, and spun at 20,000 × *g* for 10 min. RNA samples were precipitated using 1× volume of isopropanol and 0.1× volume 5 M NaOAc pH 5.2 overnight at −80°C. The RNA samples were centrifuged at 20,000 × *g* for 1 h at 4°C. Pellets were washed with cold 80% ethanol and centrifuged for 10 min at 20,000 × *g*, air-dried, and resuspended in 10 mM Tris-HCl (pH 7.0).

The RNA-seq libraries were prepared using 5 µg of total RNA of each sample. Ribosomal RNAs were removed by riboPOOLs (oligos designed for the removal of *Caulobacter crescentus* rRNAs from siTOOLs Biotech) and library construction was performed according to the protocol in reference (61). RNA-seq samples were stripped of adapter sequences using a custom Python script, reads were aligned to rRNA using bowtie, and the unaligned reads were then aligned to the genome using bowtie. RNA levels were calculated for each sample using Reads per kilobase per million (RPKM), and half-lives were calculated from the time series using Rif-Correct (62).

To estimate bulk mRNA decay rates, the total number of mRNA reads was divided by the total reads for each time point, yielding the fraction of mRNA. The fraction of mRNA across all the time points was then fit to an exponential decay equation to calculate the bulk half-life. Bulk half-lives were then subjected to a two-tailed *t*-test of unequal variance.

### Immunoblots

To confirm the fusion of the FLAG epitope to RhlB, the strain containing the FLAG-RhlB construction in the pBV-MCS4 vector was grown in PYE containing gentamycin at 30°C until OD_600_ = 0.3, when vanillate (0.5 mM) was added and then incubated for further 2 h. A *C. crescentus* strain containing a FLAG-RNE fusion (28) was grown in plain PYE medium and used as a control. Proteins were precipitated with cold trichloroacetic acid 12.5% (Sigma), resuspended in LDS Bolt 1× sample buffer (Invitrogen), and boiled for 10 min before electrophoresis, as described below.

To detect Rho and Fur proteins, *C. crescentus* NA1000 and Δ*rhlB* strains were grown in PYE at 30°C until OD_600nm_=0.4–0.5 and transferred to 10°C for 2 h. Aliquots were taken right before cold incubation and immediately after the 2-h treatment. The OD_600nm_ was normalized to 0.4, and then cells were pelleted, resuspended in 50 µL lithium dodecyl sulfate (LDS) Bolt 1× sample buffer and boiled for 10 min before transferring to ice and centrifuged at 12,000 rpm for 3 min. Supernatants were applied to a denaturing Bolt 4%–12% Bis-Tris Plus (Invitrogen) gel in 2-(N-morpholino)ethanesulfonic acid (MES) buffer and submitted to electrophoresis (150 V for 30 min). Proteins were then transferred to a nitrocellulose membrane at 250 mA for 1 h and the immunoblot was conducted as the method described previously (63). To detect FLAG-RhlB, the membrane was incubated with the primary monoclonal anti-FLAG M2 antibody (Sigma) at a 1:20,000 dilution in TBSTT (TBS (Tris buffered saline), 0.03% Tween 20, 0.02% Triton X-100) for 2 h with agitation at 4°C. To detect Rho and Fur, membranes were incubated with the primary anti-Rho (64) and anti-Fur (39) polyclonal sera at 1:1,000 and 1:50 dilutions, respectively, in TBSTT for 2 h with agitation at 4°C. After washing, the membranes were incubated with the secondary antibody (anti-mouse or anti-rabbit IgG conjugated with ALEXA-fluor 488, Invitrogen) diluted in TBS with 5% powdered milk for 1 h in the dark at 4°C. After washing, fluorescence was detected in a transilluminator UV (ChemiDoc XRS + System, Bio-Rad Lab).

### Sequence data analyses

RNA-seq data were processed using the frtc pipeline (available at https://github.com/alanlorenzetti/frtc/) (65 – 67), and differential expression analysis using DESeq2 (37) and custom scripts available at https://github.com/alanlorenzetti/ccrescentus_RNASeq_analysis. This involved using the hypergeometric distribution to determine significant overrepresentation within the target gene sets. To account for multiple hypothesis testing, the Benjamini-Hochberg method was employed for *P*-value adjustment. Integrative Genomics Viewer (68) was used to visualize and integrate the data. The Clusters of Orthologous Groups of proteins category enrichment analysis was carried out as in reference (32).

HITS-CLIP data were analyzed using the peak-caller Piranha (69). First, we merged the BEDgraph coverage replicate files using BEDtools (70) and Bash command lines. Then, we converted the coverage BEDgraph files into BED files, with the BED score column as the read count for the interval. We ran Piranha using the −s and −z 5 parameters, considering the RhlB file as the response file and the control as a covariate file. Since we were interested in the transcripts to which RhlB was bound and not necessarily in each peak, we filtered the results to get the genes to which there was at least a sense peak associated. To evaluate the significance of overlaps between RhlB-bound transcripts and differentially expressed genes, we used the hypergeometric test (File S1).

Fragmentation was indirectly inferred by the number of aligned reads representing all sorts of fragments. To avoid mathematical difficulties in normalizations, for this specific analysis, we used an undersampling approach setting artificially the sequencing depth to 90% of the number of uniquely aligned reads from the less sequenced library and randomly sampling this number of reads from all libraries equally. From the resampled BAM alignment files of paired-end RNA-seq experiments, we generated filtered files containing only paired *R*2 and unpaired *R*2 reads to account for the 5′ end of the sequenced fragments using SAMtools (71). Then, we calculated the coverage of 5′ positions from the filtered files or directly from the resampled BAM files (single-end RNA-seq experiments). The accumulated counts of how many reads start in each genomic position along the whole genome coordinate space are named 5′ profile. We merged the respective profiles of the replicates, generating one overall profile for each strain. The 5′ profile indicates a differential overall fragmentation level. To assess excess fragmentation based on the 5′ profile, we calculated the proportion of 5′ counts *P*_i_ = mutant_i_/(mutant_i_ + wt_i_) for each i genomic position. For every position in the genome, *P* = 1 means zero counts in the wild-type strain, *P* = 0 means zero counts in the mutant strain, and *P* = 0.5 means both strains have the same number of counts. Since these counts are high, a continuous Gaussian model was imposed as an approximation with variance pooled from all *P* except our biologically relevant targets, *P* = 0 and *P* = 1, assuming them as the background noise informing the model. A Bayesian Decision Theory approach was used to compare the statistical significance of the difference between *P* = 0 and *P* = 1 counts (72). Namely, we calculated the BER and declared BER < 0.01 as significant. All codes used for this analysis are available at https://github.com/bpicinato/ccrescentus_fragmentation_drhlB.

## Data Availability

All raw data of RNA-seq and HITS-CLIP data of this study are publicly available at NCBI’s Sequence Read Archive (SRA) under the BioProject accession numbers PRJNA954892 and PRJNA714975 (32). The mRNA half-life profiling data sets of wt and delta *rhlB* are available at NIH GEO data accession number GSE228592. The *P. aeruginosa* transcriptome data are available at NCBI’s SRA under BioProject PRJNA702527.

## SUPPLEMENTAL MATERIAL

Tables S1 and S2, Fig. S1 to S6 spectrum.01934-23-s0001.pdf Supplemental material.

Table S3 spectrum.01934-23-s0002.xlsx Differentially expressed genes between rhlB mutant and wt.

Table S4 spectrum.01934-23-s0003.xlsx Genes with differential mRNA decay in the rhlB mutant.

Table S5 spectrum.01934-23-s0004.xlsx RhlB-bound RNAs.

ASM does not own the copyrights to Supplemental Material that may be linked to, or accessed through, an article. The authors have granted ASM a non-exclusive, world-wide license to publish the Supplemental Material files. Please contact the corresponding author directly for reuse.
