# Supplementary material for: The DEAD-box RNA helicase RhlB is required for efficient RNA processing at low temperature in Caulobacter
Source: Microbiol Spectr. 2023 Oct 18;11(6):e01934-23. doi: 10.1128/spectrum.01934-23 (PMC10715135; doi:10.1128/spectrum.01934-23)
Supplement: Tables S1 and S2, Fig. S1 to S6 — Supplemental material. [file spectrum.01934-23-s0001.pdf]

SUPPLEMENTAL MATERIAL for manuscript:

The DEAD-box RNA helicase RhlB is required for efficient RNA processing at low temperature in *Caulobacter*

Hugo L. de Araújo, Beatriz A. Picinato, Alan P.R. Lorenzetti, Nisansala S. Muthunayake, I.W. Rathnayaka-Mudiyanselage, Naara M. dos Santos, Jared Schrader, Tie Koide, Marilis V. Marques

Table S1: Strains used in this work

	Strain	Description	Reference
Caulobacter crescentus	NA1000	Wild-type strain	1
	Δ*rhlB*	Strain with deleted RNA helicase gene *rhlB*	2
	Δ*rhlB* pBV FLAG-RhlB	Strain with deleted *rhlB* gene complemented with FLAG-*rhlB* in the pBV-MCS4 vector	This study
	Δ*rhlB* pBV RhlB	Strain with deleted *rhlB* gene complemented with *rhlB* in the pBV-MCS4 vector	2
	NA1000 FLAG-RNE	Strain with a chromosomally-encoded FLAG-RNase E	3
	Δ*fur*	Strain with deleted *fur* gene (Ferric Uptake Regulator)	4
	rhlE::Tn5	Strain with Tn5 insertion in the *rhlE* gene	5
E. coli	DH10β	Cloning strain	6
	S17-1	Conjugative strain	7

Table S2: Primers used in this work

qRT PCR primers

Entry code	Primer sequence
**CCNA_00028**	Fw: 5’ CGCCTATACCAGCGTCAACC<br>Rv: 5’ CTCTTCGACACATGGTAGGC
**23S (CCNA_R0066)**	Fw: 5’ CGAATGGGGAAACCCACCTT<br>Rv: 5’ CAGTTCCCCGGGTTTGCTTA
**16S (CCNA_R0069)**	Fw: 5’ TGGTTCGGAACAACTCAGGG<br>Rv: 5’ GCCTTGGTGAGCCTTTACCT
**RibD (CCNA_00929)**	Fw: 5’ ATGCGGCTGGAGAGACCC<br>Rv: 5’ CATGGTGGTTGCAGGGCT
**GcvT (CCNA_03465)**	Fw: 5’ ATTCGATGCCCGTGCAGTA<br>Rv: 5’ AGACCACCTTCTCGAAGCTCT
**CCNA_00422**	Fw: 5’ TACGACGTCGCCATCCACTAC<br>Rv: 5’ GTCTCGTCCTCGTCCGAAAGG
**RNase E (CCNA_01954)**	Fw: 5’ CCAAACCCGCAAACAGCTTC<br>Rv: 5’ CTGGTAGTAGTCGGGGTGGA
**Fur (CCNA_00055)**	Fw: 5’ GCCATCGATCCGCACATCTC<br>Rv: 5’ TCGATGATGCCGCTTTCCTC
**CCNA_02277**	Fw: 5’ ATTCACGCCGACCGCAAATC<br>Rv: 5’ CGTCAGGTTGAAGACGCCTG
**CCNA_03065**	Fw: 5’ CCAAGCTCTATCTGCGCTATCC<br>Rv: 5’ CGCTGGTCGTCGTCGAACTT
**CCNA_03175**	Fw: 5’ GTCCATAGGACGTCTGCCG<br>Rv: 5’ GTCGCGATGTCCCAGACGAT
**CCNA_02621**	Fw: 5’ GGCTGGCTGATGCAACTT<br>Rv: 5’ CAAGGCCAGTTCCTTCGAC
**CCNA_01789**	Fw: 5’ TCATCGGCTTTTGCGAGCTG<br>Rv: 5’ GGGCGCTTAAGGCCTCCTG
**CCNA_02781**	Fw: 5’ TAGCGGGGGCTATAAAGTGG<br>Rv: 5’ CAGTTCGAAGCTCTGGCTTG

**CCNA_02274**	Fw: 5’ CGGACATGACGCCTGAAAAG<br>Rv: 5’ GCCGGAATAATCGAACTCGG
**CCNA_03157**	Fw: 5’ ATGTACTGGGTCAACGCCAA<br>Rv: 5’ CTTCGATGACGGCGGTGTAG
**RpoH (CCNA_03195)**	Fw: 5’ CCGACCACCCTGGAAGAAC<br>Rv: 5’ CTTCTCGAACGCGCGGAC
**CCNA_2785**	Fw: 5’ GAGAAGGTGAAGCTCCCTAGC<br>Rv: 5’ GACCGAGTTCGACGATGTGA
**CCNA_01908**	Fw: 5’ GACTTGGCTACCGGATCTTC<br>Rv: 5’ CGGATAGGCCCAGGGATAG
**CCNA_02792**	Fw: 5’ CTCAACCACGTCGGTGACCT<br>Rv: 5’ AACCGTAGGCCAGATTGAGG
**CCNA_02807**	Fw: 5’ CGTGTCGGTTCCTGAAGAGG<br>Rv: 5’ GTGAGGCCCGAAAGGATCTC
**CCNA_01206**	Fw: 5’ CGTCTTCATGCCCTCGTACC<br>Rv: 5’ ATCAGAGCCGCAAGCGTCAT
**CspA (CCNA_02997)**	Fw: 5’ CCGTGAAGTGGTTCAACTCC<br>Rv: 5’ CTGACCTTCGTTCGTTCAGCGAG
**CCNA_01792**	Fw: 5’ CGTTCATCGAGGACAAGGACAC<br>Rv: 5’ CAGGATCTGCTTGCCGTTGTA
**CCNA_01440**	Fw: 5’ GCGGCATCAAGGAAACCAC<br>Rv: 5’ CACCGTTGTTGTAGATGAGCA
**CCNA_01098**	Fw: 5’ GACTTGGCTACCGGATCTTC<br>Rv: 5’ GGATAGGCCCAGGGATAG
**CCNA_01503**	Fw: 5’ TGGATTTGCGTCAACTCGTG<br>Rv: 5’ GACCGAGTTCGACGATGTGA
**Rho (CCNA_03876)**	Fw: 5’ GTCGAGAACGCCAACTCCAT<br>Rv: 5’ CGAGGGTCTTCAGGATCGC

FLAG-RhlB construction	
**Primer name**	**Primer sequence**
FLAG-RhlB Fw	5' GCGAGGAAACGCATATGGACTACAAGGACGACGACGACAAGCTGAATTCACCGACCT 3'
FLAG-RhlB Rv	5' AGGTCGGTGAATTCAGTCTTGTCGTCGTCGTCCTTGTAGTCCATATGCGTTTCCTCGC 3'

Figure S1

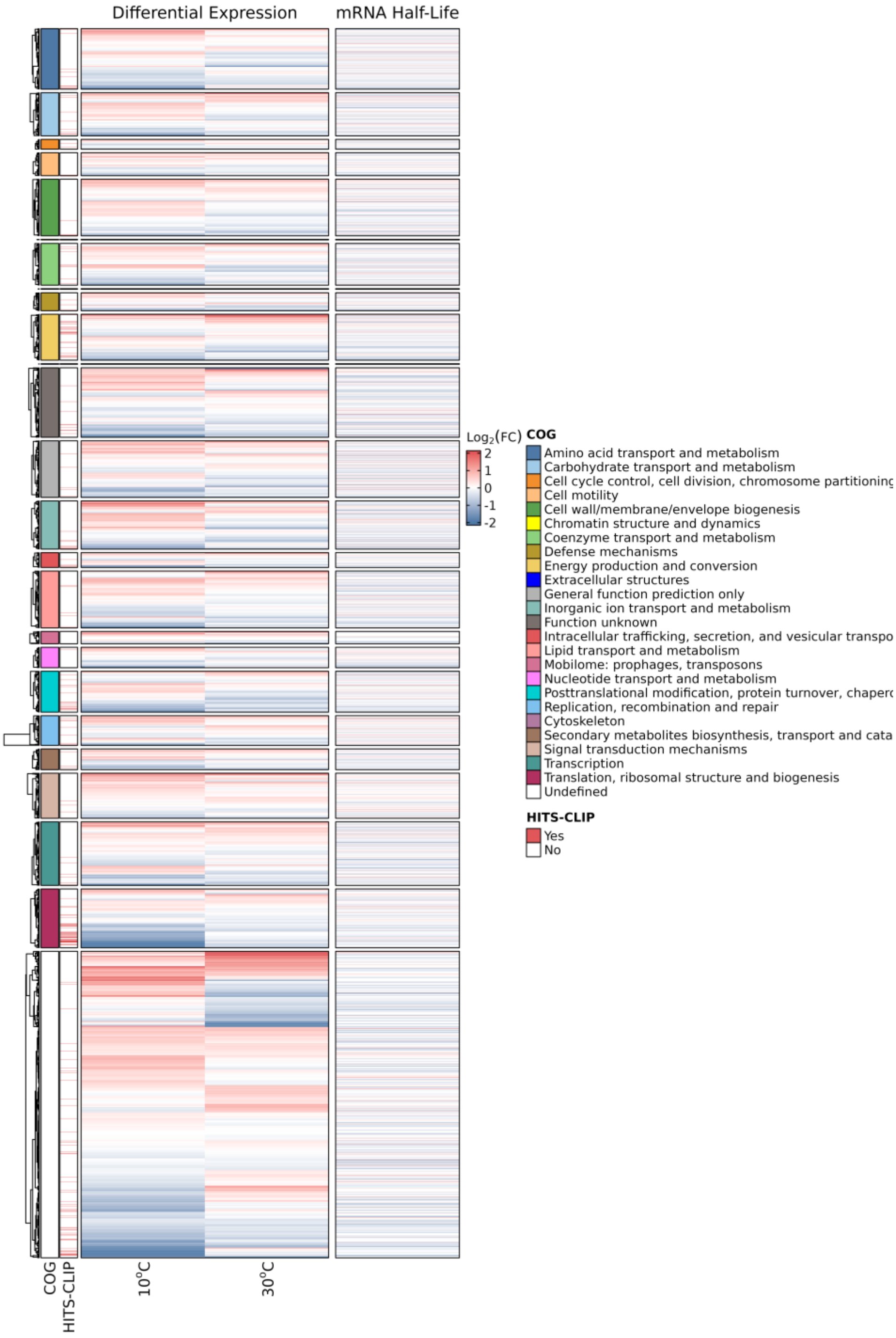

Figure S1. Global expression, decay and RhlB binding analyses of all *C. crescentus* genes. Heat map shows the $\log_2$ fold-change of the gene expression ratio between the *rhlB* mutant and the wt strain determined by the RNAseq analyses at 30°C or at 10°C. The mRNA half-life of each gene was determined after rifampicin treatment and the $\log_2$ fold change of the RNA amount ratio of *rhlB* mutant relative to the wild-type is shown in the heat map. mRNAs that putatively bind to RhlB (enriched in the FLAG-RhlB vs RhlB strains) are indicated in red in the HITS-CLIP column. Figure made using R package ComplexHeatmap (8).

Figure S2

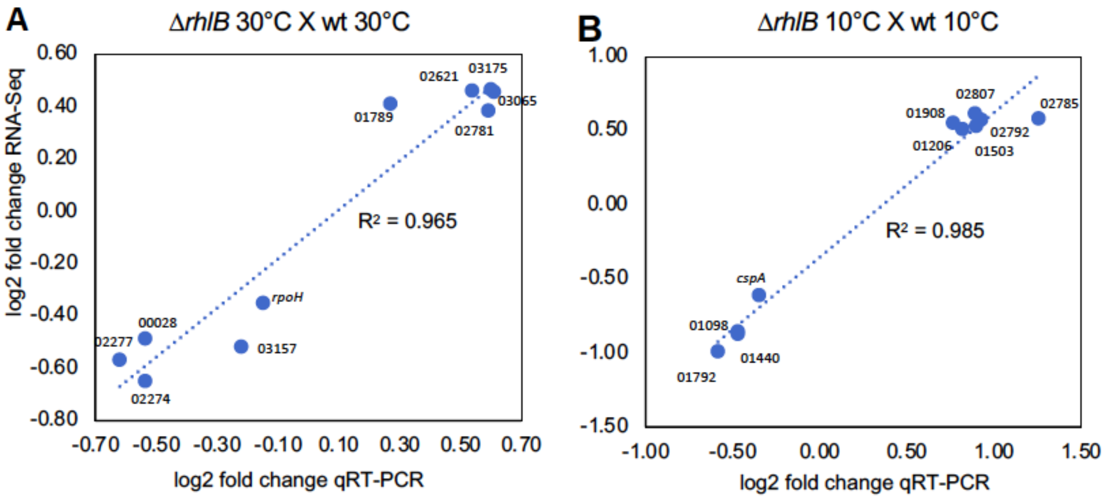

Figure S2. Validation of RNA-Seq data by RT-qPCR analysis. Relative expression was determined by RT-qPCR from total RNA of cells incubated at 30°C until mid log phase, and after 2h cold-shock at 10°C on the Δ*rhlB* strain relative to the wild type (wt, NA1000 strain). The $\log_2$ fold change in RT-qPCR data were plotted against the $\log_2$ fold change obtained from RNA-Seq data. The expression levels for selected genes were plotted, and the identification of each gene is indicated.

Figure S3

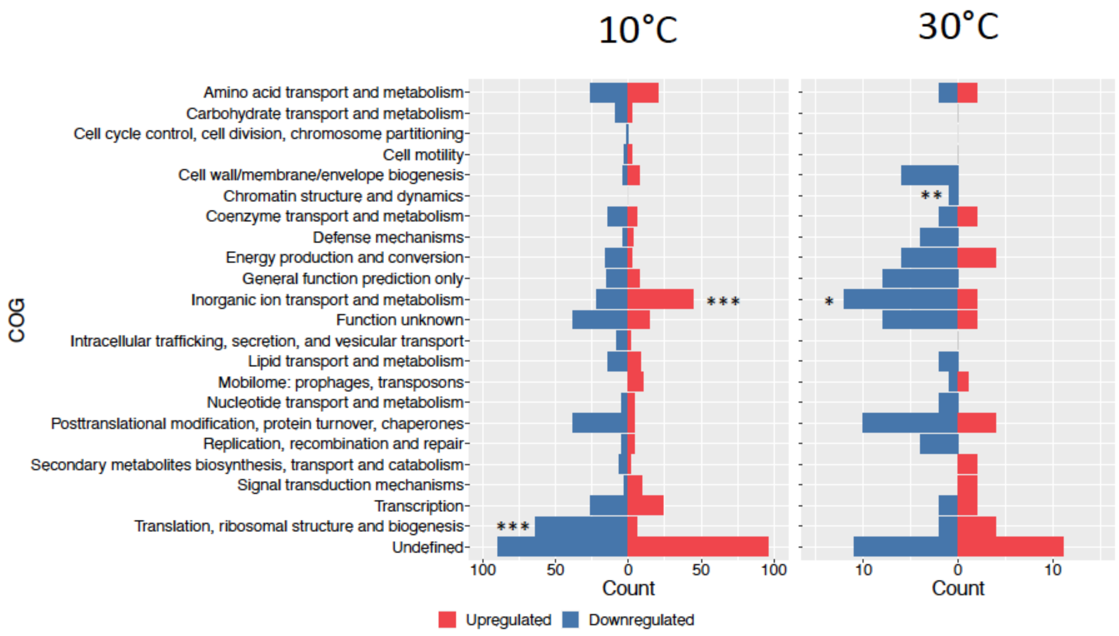

Figure S3. DEG functional characterization. Graphical representation of the functional categories enriched among the DEG in both RNA-Seq analyses (30ºC and 10ºC). The total number of genes up- or downregulated identified as belonging to each category of the Orthologous Group of Proteins (COG) was analyzed as to verify whether categories were significantly enriched against the total number of DEGs. The categories defined to be overrepresented in each analysis are those with the Benjamini-Hochberg adjusted p-value < 0.05, as indicated: * adjusted p-value <0.05; ** adjusted p-value <0.01; *** adjusted p-value <0.001.

Figure S4

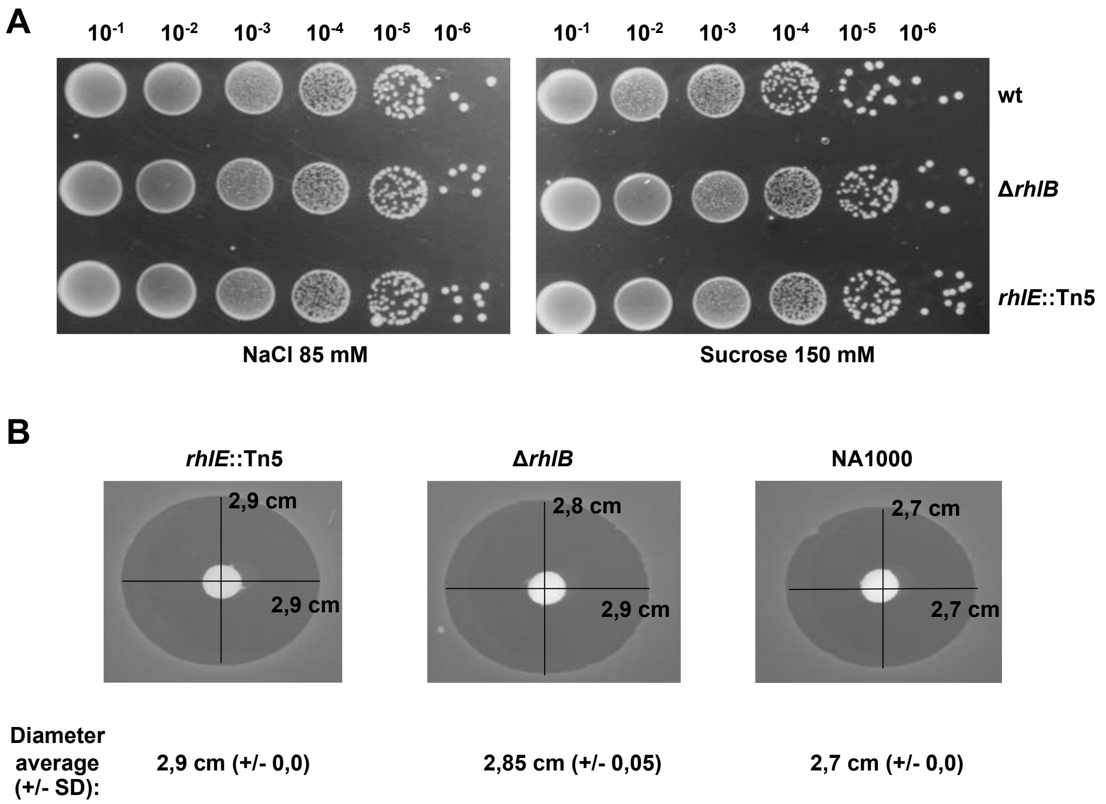

Figure S4. Viability and sensitivity tests of the NA1000, *rhlE*::Tn5 and Δ*rhlB* strains to osmotic and oxidative stress. A) The wt (NA1000), Δ*rhlB* and *rhlE*::Tn5 strains were grown in PYE medium until mid-log phase and 85 mM NaCl or 150 mM sucrose were added and further incubated for 6 h at 30°C. Serial dilutions of the cultures were plated in PYE using biological duplicates and further incubated at 30°C. B) Sensitivity to oxidative stress was determined by measuring the halo of growth inhibition by H_2O_2. 0.5 ml of each midlog culture was spread onto PYE plates and a disk containing 10 ml of 3% H_2O_2 was placed over the plates. After incubation at 30°C for 72 h the diameters of the halos were measured. The average of the halo diameters +/- the Standard Deviation (SD) are shown below the respective strain. The results are representative of two tests using biological duplicates.

Figure S5

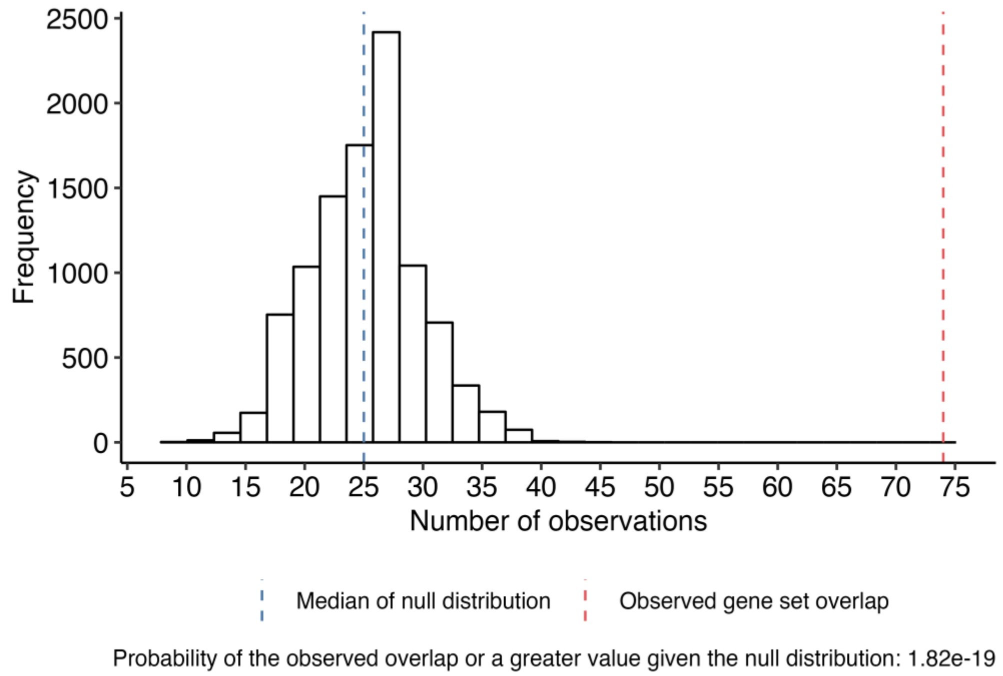

Figure S5. Overlap significance between the RNAs identified in the HITS-CLIP experiment and DEGs at 10°C. A significant overlap (74; p = 1.82e-19) was observed between the set of genes bound to RhlB (216) and those differentially expressed at cold temperatures (10°C; 478). For that, we generated a simulated null hypergeometric distribution (white bars) using the parameters from our empirical data. Then, we computed the probability of finding the observed overlap (red dashed line) or a more extreme value within this distribution.

Figure S6

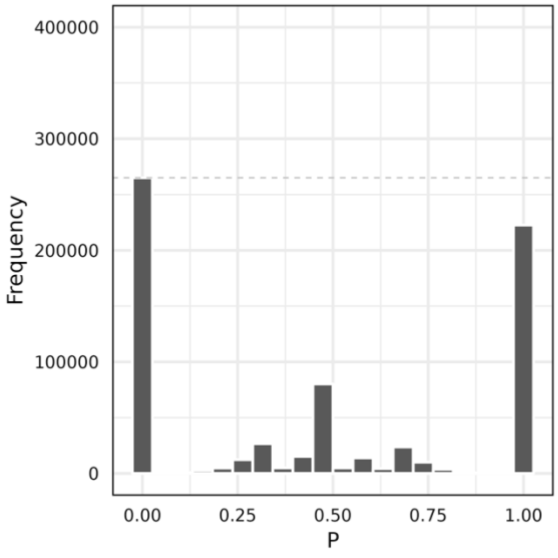

Figure S6: Histogram of values of P for *C. crescentus* Δ*iscR* mutant. The fragmentation analysis was carried out using *C. crescentus* RNA-Seq data of a transcription factor (*iscR*) mutant strain in relation to its wild-type control. The proportion P of 5’ end counts of sequenced fragments in the Δ*iscR* mutant strain relative to the total of 5’ ends counts of both in the mutant and wild-type strains was calculated for every genomic position. Genomic positions where P = 0, no reads starting at that position in the mutant, and P = 1, no reads starting at that position in the wild-type strain, respectively. The Δ*iscR* RNA-Seq library was prepared and sequenced in the same way as the Δ*rhlB* sequencing library prepared in this study. We do not observe significant fragmentation excess at the genome level for this mutant strain relative to control (BER=0.25).

References

1. Evinger M, Agabian N. 1977. Envelope-Associated Nucleoid from *Caulobacter crescentus* Stalked and Swarmer Cells. J Bacteriol 132(1):294–301.

2. Aguirre AA, Vicente AM, Hardwick SW, Alvelos DM, Mazzon RR, Luisi BF, Marques MV. 2017. Association of the cold shock DEAD-box RNA helicase RhlE to the RNA degradosome in *Caulobacter crescentus*. J Bacteriol 199(13).

3. Hardwick SW, Gubbey T, Hug I, Jenal U, Luisi BF. 2012. Crystal structure of *Caulobacter crescentus* polynucleotide phosphorylase reveals a mechanism of RNA substrate channelling and RNA degradosome assembly. Open Biol 2(4):120028.

4. da Silva Neto JF, Braz VS, Italiani VCS, Marques MV. 2009. Fur controls iron homeostasis and oxidative stress defense in the oligotrophic alpha-proteobacterium *Caulobacter crescentus*. Nucleic Acids Res 37(14):4812–4825.

5. Mazzon RR, Lang EAS, Braz VS, Marques MV. 2008. Characterization of *Caulobacter crescentus* response to low temperature and identification of genes involved in freezing resistance. FEMS Microbiol Lett 288(2):178–185.

6. Hanahan D. 1983. Studies on transformation of *Escherichia coli* with plasmids. J Mol Biol 166(4):557–580.

7. Simon R, Priefer U, Pühler A. 1983. A broad host range mobilization system for in vivo genetic engineering: transposon mutagenesis in gram negative bacteria. Biotechnol 1:784-791.

8. Gu Z, Eils R, Schlesner M. 2016. Complex heatmaps reveal patterns and correlations in multidimensional genomic data. Bioinformatics 32(18):2847–2849.
